# Chloroform or Ether

**Published:** 1862-02

**Authors:** 


### CHLOROFORM OR ETHER.

Reprinted from the *London Lancet.*

Fifteen years have just been completed since the first capital operation was performed upon a patient who had been subjected to the influence of an anæsthetic agent. This was done by Professor Hayward at the Massachusetts General Hospital on November the 17th, 1846. The operation performed was amputation above the knee in a female. Previous to this undertaking, Dr. Morton had extracted a tooth, and Dr. Warren had removed a nævus from the face, under the same circumstances. The dates of these operations were September 30th and October 16th, preceding the time of Professor Hayward's venture. The anæsthetic agent employed upon each occasion was sulphuric ether. Since that period until the present time anæsthetics have been in constant use, and for all sorts of purposes where the induction of insensibility to pain is desired, and the arrest of convulsive and spasmodic movements sought for. We have seen it employed in lithotomy, in tetanus, in cases of poisoning by strychnia, in whooping-cough, and in parturition. Teeth are removed without pain by its aid, and we have witnessed the most urgent vomiting of pregnancy arrested by its application along with that of opium to the epigastrium. The uses of anæsthetics, indeed, are manifold. It is not surprising, then, that several agents should have been recommended as endowed with superior soothing and benumbing powers. Of them, however, ether and chloroform have alone kept the field. Of amylene we do not hear anything now, and we presume that keroselene, the last candidate for favor, will soon be forgotten. Whether the first two will continue to occupy the field, or whether one will finally displace the other, and which will be the conqueror, are points upon which it would be now premature to decide. Ether is not employed as an anæsthetic agent to any extent in Great Britain nor in many parts of Continental Europe. Here chloroform is used. But, upon the other hand, ether is habitually exhibited at Lyons and Naples, and is the only anæsthetic administered in the principal hospitals of the United States of America. And what are the reasons given for the adoption of each particular agent by its employers? "We use ether in preference to chloroform," say the advocates of the former, "because it never kills." "We prefer chloroform to ether," urge the partizans of other, "because it is quicker and surer in its action, more agreeable to breathe, and more portable to carry." "We deny that these asserted advantages of chloroform are so prominent as you maintain. Nor is ether so decidedly innocent as you affirm." "Ether," replies Professor Hayward, "is, I am confident, a perfectly safe anæsthetic agent. I have not been able to find any well-attested case of death from its inhalation. There may have been such, but they have never come to my knowledge, though I have taken unwearied pains to obtain information on this point.—I have given it in several hundred cases, and witnessed its exhibition by others in as many more. I have administered it to infants not three weeks old, and to persons more than three-score years and ten, and have never in a single instance seen an alarming or distressing effect produced by it." The surgeons of Lyons declare, "that since the adoption of ether in place of chloroform, the necrology of anæsthesia has not received an additional instance" in their city. Signor Palaschiano, of Naples, maintains it to be "infinitely safer than chloroform;" and in Mr. Erichsen's work it is affirmed of ether that "no death has yet resulted from its use."

As we before stated, fifteen years have past since the contest began, and it cannot be considered to be yet settled. Some nine or ten months since we laid before our readers the intention of the Boston Society for Medical Improvement to appoint a Committee "to investigate the alleged deaths from the inhalation of sulphuric ether, and to report thereon." The desire of our American brethren to arrive at a conclusive judgment upon an important question connected with anæsthetics was widely made known to the profession, in order that the latter might give all possible assistance. The Committee has just issued its Report.* It commences, after a few prefatory remarks, with the following preamble:—

"When the subject of chloroform first came under discussion, its dangers were commented upon, and even then freely acknowledged. It had not been two months introduced, when 'a well developed girl of fifteen' died from its admininstration for the evulsion of a toe-nail, 'the process of inhalation, operation, and death, not having occupied more than 5 minutes.' Since that time deaths from its use have repeatedly occurred. On the other hand, fatal results from ether, although still fig-

* Report of a Committee of the Boston Society for Medical Improvement on the Alleged Dan- which accompany the Inhalation of Sulphuric Ether. pp. 36. Boston, 1861.

uring in the statistics of mortality from anæsthetics, are everywhere admitted to be infrequent. Indeed, the opinion has been expressed by various authorities both in America and Europe, that a death really attributable to the inhalation of sulphuric ether is yet to be reported. The correctness of this opinion has, however, been repeatedly denied, and the strong conviction of the absolute safety of this agent which exists in some localities in this country is thought to have its foundation rather in the desire that the fact might be established, than in the proof that it was so. Of course no one intends to say that a person cannot be killed by ether. The inhalation of its vapor without a sufficient admixture of oxygen destroys life by asphyxia. This may happen, and unfortunately has happened, but such an event cannot be laid to the anæsthetic, since in such a case it is the method of administration, and in no sense the ether which causes the fatal result. It is the purpose of this Report to solve the doubt just implied with regard to the absolute safety of sulphuric ether, and to investigate the dangers of its use as compared with chloroform."—(p. 3.)

After having considered what conditions and precautions are necessary in bringing about a state of insensibility by the employment of ether, and what phenomena of etherization have an apparent or real danger, the Committee next undertake to prove that, these conditions being fulfilled, "sulphuric ether is of all anæsthetic agents alone worthy of unlimited confidence." This is a simple and bold statement, and could it be received without demur—which we think it cannot, after perusal of the Report before us,—chloroform, the anæsthetic most in vogue, would gradually be displaced. The Boston umpires maintain that, with unequalled facilities to examine the literature of the subject under discussion, with all the chief foreign and American Journals at hand, and the results of a most extensive distribution of circulars, no case of which they have knowledge can be cited as *unquestionably* and *unavoidably* fatal from the inhalation of pure sulphuric ether. The whole number of alleged deaths from this agent collected by the Committee is forty-one, and some details of each case are given in the appendix; references are also made to other instances of death stated to have been in any way connected with the inhalation of ether. Now all these examples are rejected by the Reporters as failing to prove that the ether was necessarily the cause of death, because not one is limited by those conditions which they consider as essential to any case of death fairly attributable to the inhalation of an anæsthetic agent. These conditions are—1st, that death should occur while the patient is actually in an anæsthetic state; 2d, that its occurrence should be inexplicable by any phenomena of disease or operation. Now out of forty-one cases detailed, sixteen survived the operation from three to sixteen days; whilst in seventeen cases, where death was immediate or nearly so, the Reporters regard the connexion between the result and the inhalation as "problematical or else manifestly absurd and unfounded, except in four instances where it is due to asphyxia, brought about by wholly avoidable causes." The remaining cases of the table "are no better able to stand the test of examination," and hence the Boston Committee have determined that their careful search "furnishes not a single conclusive case of death from the proper inhalation of sulphuric ether" (p. 12).

We have not the least doubt but that the Boston Committee has believed itself justified in coming to this decision. We, upon the contrary, think it has mystified itself in a maze of special pleading and assumption. The same desire to seek for a more recondite cause of death than that by ether, applied to the fatal cases from chloroform, would equally acquit this agent also, and explain away half of its alleged mortality. The details of many of the cases given in the Report are, no doubt, of such a kind as to show that the inhalation of ether was probably not unavoidably and unquestionably the cause of death unhelped by contingent circumstances. But may not the same thing be said of the recorded deaths from chloroform? Further, the perusal of other cases cannot but lead the unprejudiced mind to think that to the ether, and the ether alone, is the fatal event to be attributed. Only let the word *chloroform* be substituted for *ether* in these instances, and the Boston Reporters would not have much hesitation in assigning the cause of death. We do not think, then, that our American *confreres* have by any means proved the perfect innocuousness of ether. They have published, however, a very interesting Report.

